# Community-Based Interventions to Improve Outcomes for Cardiovascular Disease [Letter]

**DOI:** 10.5888/pcd11.140390

**Published:** 2014-10-09

**Authors:** Marta A. Miyares, Kyle A. Davis

To the Editor:

We applaud Oldenburg and colleagues for the successful implementation of a community-based intervention to curtail cardiovascular disease (CVD) (1). The authors promote the increased use of aspirin for primary prevention (the aim of which is to reduce the risk for a first heart attack or stroke) among people who meet the criteria established by the 2009 US Preventive Services Task Force. However, although their intervention was effective, we believe the use of aspirin for primary prevention should be approached with caution: trials of aspirin use in primary prevention have demonstrated only a modest improvement in clinical outcomes.

Consistent with various meta-analyses, Raju and colleagues showed that aspirin was associated with a slight reduction in overall mortality (relative risk, 0.94 [95% confidence interval, 0.88–1.0]); absolute risk reduction, 0.09%) but had no effect on CVD mortality (2). In another investigation, a reduction in the risk for negative CVD outcomes was not demonstrated when low-dose aspirin was used for the primary prevention of atherosclerotic events among patients with type 2 diabetes (3).

Review of these and other trials led the Food and Drug Administration in May 2014 to deny a request from Bayer Healthcare to change aspirin’s label to indicate use for primary prevention of CVD. The agency stated that the use of aspirin for primary CVD prevention is not supported by the evidence and that “there are serious risks associated with the use of aspirin, including increased risk of bleeding in the stomach and brain, in situations where the benefit of aspirin for primary prevention has not been established” (4). In the design of the study by Oldenburg and colleagues, no consideration was extended to patient factors that would deem the use of aspirin inappropriate because of an increased risk of bleeding or other contraindications (1). The US Preventive Services Task Force is reviewing the role of aspirin in people without known CVD to assess changes in rates of myocardial infarction (MI), stroke, death from MI or stroke, and all-cause mortality, potentially further relegating aspirin to a lesser role in the primary prevention of CVD.

Although the community-based program described by Oldenburg and colleagues was effective in increasing aspirin therapy among patients at risk for CVD, implementation of established therapies such as statins may prove to be more valuable. In a comparison of aspirin and statins for the primary prevention of CVD, Dietrich and Davis concluded that statins have a better risk-to-benefit profile; in their evaluation, aspirin therapy resulted in the absolute reduction of major CVD events by 0.1%, whereas statin therapy resulted in an absolute reduction of 1% to 2% (5). An assessment by Austin and colleagues of the effects on mortality of underprescribing statins showed missed opportunities in the secondary prevention of MI. In their study of 7,285 survivors of acute MI (AMI) discharged from 102 hospitals in Ontario, Canada, they concluded that modest increases in statin prescribing among patients least likely to receive a prescription at discharge could lead to decreases in post-AMI mortality at the population level (6). Furthermore, because the risk for gastrointestinal bleeding and intracranial hemorrhage (2 of the most serious adverse events associated with aspirin) is 100 times the risk for rhabdomyolysis and hemorrhagic stroke (2 of the most serious adverse events associated with statins), targeting statin therapy for intervention seems prudent (5).

Although the study by Oldenburg and colleagues highlights the potential success of community-based interventions in promoting the primary prevention of CVD, we advocate the use of therapies that have better, proven effects than aspirin has.

**Marta A. Miyares, PharmD**
**Kyle A. Davis, PharmD**
Jackson Memorial Hospital
1611 NW 12th Ave
Miami, FL 33136-1096
